# Mark Bailey, *After the Black Death: Economy, Society, and the Law in Fourteenth-Century England. The Ford Lectures for 2019*

**DOI:** 10.1093/shm/hkab058

**Published:** 2021-05-18

**Authors:** Daniel R Curtis

**Affiliations:** Erasmus University Rotterdam, The Netherlands

This book reasserts the importance of the Black Death in its capacity to transform societies; in the process creating ‘structural change’ in late-medieval England—that is modification of institutional arrangements with the potential for lasting and persistent economic effects. More specifically, the book’s main contributions are twofold. First, it shows the importance of factor markets (in land, labour and capital) and commodity markets in England after the Black Death—which were less influenced by manorial control or interference than previously assumed. Indeed, manorial lordship is presented as less of a constraining force on English economic development compared to a previous view in the historiography. Secondly, it reconsiders the temporal dynamics of the economic impact of the Black Death in England, arguing instead for the formative importance of the third quarter of the fourteenth century (i.e. the aftermath of the Black Death itself), rather than a period after the 1370s. More generally and ambitiously, the book suggests that the structural change provoked by the Black Death was significant enough to set in motion certain processes that led to England becoming an eventual economic forerunner—thereby contributing to the Little Divergence debate (a debate on when and how the economies of Northwest Europe forged ahead of other parts of Europe).

Perhaps unexpected, Bailey succeeds much more with the specific contextualised contributions for medieval England than with the general broader one. The book is erudite and nuanced—unsurprising to anyone acquainted with classics of English medieval economic history such as *A Marginal Economy*, *Modelling the Middle Ages* and *The English Manor*.^1^ The subject material is complex, but Bailey does a laudable job in unpacking complicated ideas, and emphasising to the reader why they are relevant or have significance. The book employs original archival material, and this is well integrated within a vast knowledge of the masses of secondary literature produced on economy and society in the period before and after the Black Death. The book convinces the reader that while pandemics can lead to structural change, the direction and nature of this change is contextually driven—and Bailey demonstrates that point by showing that the qualities of factor markets were dependent on the social and demographic contexts in which they were embedded.^2^ One of the most valuable aspects to this book is the explicit integration of the law into social and economic history, and Bailey shows through the English experience that the law was not irrelevant or even inevitably deleterious to the welfare of peasants and laborers—that is merely a tool of elites—but could, at times, facilitate and protect the choices of ordinary people.

There are, of course, some limitations to this book—issues that are recurring features of scholarship from British medieval economic history. One wonders what is the added value of framing the book as a contribution to the Little Divergence debate, when the substantive empirical material only corresponds to one place within the divergence—England. To coin a recent *Economic History Review* title: ‘What can we learn from a race with one runner’?^3^ Indeed, while Bailey rather implicitly has suggested ‘continental scholars’ (Bailey’s term) have effectively misunderstood or misinterpreted certain aspects of English medieval history (pp. 10–12, 24), his own book provides a vast array of evidence on complex aspects of property and tenurial structure, laws and legal codes, and factor markets in England, yet this is placed against a few sparse references to GDP estimates, real wages and marriage patterns in a select few other areas of Europe.^4^ Towards the end of the book, Bailey calls for greater integration of econometric modelling and fine-grained micro-histories, as well as ‘more comparative studies’ (p. 285), and yet he has done neither integrating of methods nor effective comparison. For example, Bailey tries to assess his nuanced and fine-grained evidence for the effect of the Black Death on the decay of serfdom in England in a broader perspective—asking why serfdom was re-entrenched or tightened in other parts of Europe (pp. 308–313). Yet, his object of comparison is restricted to a few references to vaguely defined areas of Central and Eastern Europe (where the temporal gap between Black Death and suggested ‘Second Serfdom’ is so large that we should critique any causal connection), while remaining silent on other pertinent post-Black Death developments such as new jurisdictional relationships between cities and their rural hinterlands in Italy, Iberia and areas of the Holy Roman Empire, and failing to mention entirely that the manor or serfdom was not even relevant for many parts of Europe—even prior to the Black Death in certain places. Put simply, Bailey convinces the reader of the impact of the Black Death on the functioning of institutions and markets in England, but the relativising of these findings leaves something to be desired.

Notwithstanding an odd reference in the endnotes (p. 175), we do not get much insight into why or how the areas and the sources have been chosen in the book—basic issues of representativeness. By employing numerous examples from different places and sources across parts of England, the book is effectively argumentation by weight of examples creating a narrative thrust rather than a systematic test and control of variables. Given that one of Bailey’s major arguments is that factor markets were ‘inefficient’ in conditions of high population pressure before the Black Death, and more efficient thereafter, surely there is a missed opportunity in not systematically testing the functioning of factor markets before and after the Black Death in two carefully selected regions with differing levels of pre-Black Death population pressure on resources? This would have wider importance as it has been shown in the broader literature that places with high pre-Black Death Malthusian pressures on resources experienced different economic trajectories after the Black Death to those that were more land abundant and labour scarce.^5^

Despite the claims for an ‘emboldened’ approach in the preface, the book is actually quite a traditional approach to medieval social and economic history. Although sporadically mentioned, Bailey does not engage that comprehensively with the major gains in plague studies in recent years such as genetics and historical climatology—unlike Bruce Campbell’s earlier ‘*The Great Transition*’.^6^ It also does not engage with the major findings in bioarchaeology—curious because many sites have been excavated in England, and have obvious relevance for any work on the post-Black Death welfare of ordinary people. It also does not integrate any original demographic material. Bailey suggests that he is providing differential social and economic outcomes from ‘the same, sudden, universal, demographic shock’ (p. 337), and yet recent literature has suggested the mortality characteristics of the Black Death, and also the severity and pervasiveness of recurring plague outbreaks, were not the same throughout Europe. The ‘demographic shock’ in the Little Divergence debate, then, is not a control variable, but a potential independent variable, that needs to be somehow separated and isolated from other variables such as differences in institutional arrangements, factor markets, and patterns of tenure. Women—and their place within the complex analysis of factor markets and inheritance practices—barely feature in the book, notwithstanding a few sporadic references, and mainly in the context of fertility and nuptiality.

Accordingly, this will be a well-read and appreciated book in medieval social and economic history quarters—especially in Britain—and one cannot deny its sophistication. Its broader appeal will likely be much more restricted on two grounds. First, the contribution to the Little Divergence debate is unclear. Secondly, plague studies more generally, and the history of the Black Death in particular, has become more global and interdisciplinary.^7^

## Footnotes

1 Mark Bailey, *A Marginal Economy? East Anglian Breckland in the later Middle Ages* (Cambridge: Cambridge University Press, 1989); John Hatcher and Mark Bailey, *Modelling the Middle Ages: The History and Theory of England’s Economic Development* (Oxford: Oxford University Press, 2001); Mark Bailey, *The English Manor c. 1200–1500* (Manchester: Manchester University Press, 2002).

2 For a broader pan-European elucidation of the same ideas: Gérard Béaur, Philipp R. Schofield, Jean-Michel Chevet and María Teresa Pérez Picazo (eds.), *Property Rights, Land Markets and Economic Growth in the European Countryside (Thirteenth–Twentieth Centuries* (Turnhout: Brepols, 2013).

3 Jeremy Edwards and Sheilagh Ogilvie, ‘What can We Learn from a Race with One Runner? A Comment on Foreman‐Peck and Zhou, “Late marriage as a contributor to the industrial revolution in England”’, *Economic History Review*, 2019, 72, 1439–46.

4 The material the English evidence is placed against amounts to a sparse handful of references from Bas van Bavel, Jan Luiten van Zanden, Tine De Moor, Sheilagh Ogilvie, Mattia Fochesato, Erich Landsteiner, Paolo Malanima, and Sevket Pamuk. Economic historians employing large datasets of quantitative evidence are curiously and indiscriminately described as ‘econometricians’, regardless of their methods or background. Scholars explicitly focusing on the role of plague in stimulating economic change or divergence such as Guido Alfani are not mentioned at all.

5 Compare: Guido Alfani and Tommy Murphy, ‘Plague and Lethal Epidemics in the Pre-Industrial World’, *Journal of Economic History*, 2017, 77, 314–43; with Carlos Álvarez-Nogal, Leandro Prados de la Escosura and Carlos Santiago-Caballero, ‘Economic effects of the Black Death: Spain in European Perspective’, *Investigaciones de Historia Económica – Economic History Research*, 2020, 16, 35–48.

6 Bruce M.S. Campbell, *The Great Transition: Climate, Disease, and Society in the Late Medieval World* (Cambridge: Cambridge University Press, 2016).

7 See the insightful Monica Green, ‘The Four Black Deaths’, *American Historical Review*, 2020, 125, 1601–31.

