# Erratum

**DOI:** 10.1093/shm/hkab065

**Published:** 2021-12-11

Mark Bailey, *After the Black Death: Economy, Society, and the Law in Fourteenth-Century England. The Ford Lectures for 2019*. Oxford: Oxford University Press, 2021. Pp. 384. ISBN 9780192599735.

By Daniel R. Curtis

*Social History of Medicine*. doi:10.1093/shm/hkab058

“Due to a copyediting error, “UK” was erroneously used instead of “England” throughout the paper. This has now been corrected online. The publisher apologises for the error"

